# Cell Death Modalities in Therapy of Melanoma

**DOI:** 10.3390/ijms26083475

**Published:** 2025-04-08

**Authors:** Meng Wang, Jia-Hui Zhao, Ming-Xuan Tang, Meng Li, Hu Zhao, Zhong-Yu Li, An-Dong Liu

**Affiliations:** 1 Department of Human Anatomy, School of Basic Medicine, Tongji Medical College, Huazhong University of Science and Technology, Wuhan 430030, China; wangmeng722@yeah.net (M.W.); u202213327@hust.edu.cn (J.-H.Z.); u202110295@hust.edu.cn (M.-X.T.); u202110311@hust.edu.cn (M.L.); zhaohu@hust.edu.cn (H.Z.); 2 National Demonstration Center for Experimental Basic Medical Education, Huazhong University of Science and Technology, Wuhan 430030, China

**Keywords:** melanoma, therapy, mechanism, cell death

## Abstract

Melanoma, one of the most lethal cancers, demands urgent and effective treatment strategies. However, a successful therapeutic approach requires a precise understanding of the mechanisms underlying melanoma initiation and progression. This review provides an overview of melanoma pathogenesis, identifies current pathogenic factors contributing to mortality, and explores targeted therapy and checkpoint inhibitor therapy. Furthermore, we examine melanoma classification and corresponding therapies, along with advancements in various cell death mechanisms for melanoma treatment. We also discuss the current treatment status along with some drawbacks encountered during research stages such as resistance and metastasis.

## 1. Introduction

Melanoma, a malignant tumor originating from melanocytes, primarily affects the skin, with sunlight exposure being a prominent risk factor [1,2]. It is an aggressive form of skin cancer characterized by high morbidity, mortality, and poor prognosis due to its highly invasive nature [3,4]. Based on prevalent mutational patterns, melanoma can be classified into four subtypes: mutant BRAF, mutant RAS, mutant NF1, and Triple-WT (wild type), with BRAF or RAS mutations being the most common drivers of melanoma development [5]. Early-stage I–II melanoma is typically treated through complete surgical excision, yielding favorable outcomes with a five-year survival rate of up to 99.4% [6]. Clinical trials of vemurafenib, dabrafenib, or pembrolizumab have shown improved progression-free survival and overall survival in patients with BRAF^V600^ mutations [7,8,9,10]. However, patients with resected stage III and IV melanoma have a high risk of recurrence [10]. Although metastatic melanoma still carries substantial mortality rates, targeted therapy involving BRAF/MEK inhibition and immunotherapy utilizing PD-1/PD-L1 inhibitors along with CTLA-4 inhibitors has led to remarkable advancements in treatment outcomes and prognosis improvement [11,12,13,14]. Targeting MEK is currently the most developed targeted strategy in NRAS-mutated melanoma while KIT inhibitors are approved for KIT-mutated cases specifically [15,16,17]. Patients with BRAF-mutated melanomas also demonstrate positive initial responses to BRAF/MEK inhibition therapies which offer considerable benefits [12]. Moreover, the most notable immunotherapies for treating melanoma involve immune checkpoint inhibition targeting CTLA-4 and PD-1 [18]. Combined with BRAF/MEK-directed therapy, these treatments have dramatically improved the prognosis for patients with metastatic melanoma [19]. However, resistance to therapy remains a significant challenge, particularly with BRAF/MEK-targeted therapies, which often have limited efficacy durations [20]. This review provides an overview of the current treatment modalities and emerging therapeutic strategies for melanoma, focusing on the molecular mechanisms underlying melanoma progression and resistance to therapy. Understanding these mechanisms is crucial for developing more effective and durable treatment options for melanoma patients.

## 2. Pathogenesis

Since the 1980s, significant efforts have been made to identify individual mutations commonly occurring in cancers, including melanoma. This has led to the development of targeted therapies tailored to the genetic profiles of patients, aiming to improve outcomes and reduce side effects by focusing on the molecular mechanisms driving the disease [21,22,23]. Approximately 5–12% of melanomas are inherited, with hereditary variants displaying unique mutational signatures compared to non-hereditary melanomas [21]. Melanoma exhibits the highest mutation burden among all cancer types [24]. Its pathogenesis involves multiple signaling pathways regulated by various genes, with numerous agents currently under development to target these pathways. The RAS/RAF/MAPK/ERK signaling pathway is predominantly activated in melanomas through NRAS or BRAF mutations [25]. KIT, a tyrosine kinase receptor which activates the RAS and PI3K pathways, is more frequently observed in melanomas originating from non-sun-exposed types [26,27]. KIT mutations are most commonly found in acral and mucosal melanomas [28]. Additionally, melanoma cases ascertained independently of family history show a much lower mutation rate [29]. Alterations in the PI3K/PTEN/AKT signaling pathway, which regulates proliferation, migration, survival, and differentiation during adult homeostasis and tumorigenesis, are also observed in melanoma tumors and cell lines [30,31,32].

The CDKN2A mutation is frequently seen in familial melanoma syndromes, although somatic CDKN2A mutations can also be found in sporadic melanomas [33]. Families with mutations in the CDKN2A gene often show a high occurrence of clinically atypical, yet benign, nevi, as well as a familial tendency towards melanoma [21].The CDKN2A gene comprises four exons (1α, 1β, 2, and 3), encoding two proteins: p16 (encoded by exons 1α, 2, and 3) and p14ARF (encoded by exons 1β, 2, and 3). Mutations in CDKN2A remain the most prevalent in hereditary melanoma [26]. In familial melanomas, up to 40% harbor CDKN2A mutations, leading to deficiencies in the p14ARF and p16INK4A proteins. These proteins serve as vital tumor suppressors, regulating the G1 checkpoint and stabilizing p53 expression [21,34]. Studies have shown that inactivation of the CDKN2A locus stimulates cell proliferation and suppresses apoptosis.

Ultraviolet radiation (UVR), particularly UVB, exerts complex and often contradictory effects on melanoma. UVR can induce DNA damage, leading to mutations that drive melanoma development. Additionally, UVR promotes the proliferation and survival of melanocytes by activating oncogenic signaling pathways, while simultaneously suppressing immune responses, thereby facilitating tumor evasion and progression [35,36]. Moreover, melanin pigment and the melanogenesis process are inherent to melanocytes and play a pivotal role in melanoma pathobiology. The presence of melanin exerts a dual influence on melanoma progression by potentially promoting melanocyte malignant transformation while also confounding therapeutic responses [37].

Several mutated genes associated with melanoma are summarized in Table 1.

## 3. Classification

According to the National Comprehensive Cancer Network (NCCN) guidelines, cutaneous melanoma is classified as melanoma in situ when confined to the epidermis or invasive when atypical melanocytes progressively invade the dermis. The traditional morphologic classification by histopathology distinguishes four major clinicopathological subtypes: superficial spreading melanoma (SSM), nodular melanoma (NM), lentigo maligna melanoma (LMM), and acral lentiginous melanoma (ALM) [52]. Less common variants include desmoplastic melanoma (DM, ~1–2% of all melanomas), amelanotic melanoma, Spitzoid melanoma, mucosal melanoma, spindle cell melanoma, and ocular (eye) melanoma (DermNet website).

Based on the degree of solar elastosis associated with melanomas on sun-exposed skin, melanomas are further stratified into low and high cumulative solar damage (CSD) groups. The low-CSD group comprises SSM, while LMM and DM fall under the high-CSD category [53]. This subtype is often found in areas frequently exposed to the sun during outdoor activities, such as the back in men and the back of the legs or calf region in women [54]. Melanoma is also linked to UV exposure from tanning beds, leading to a notable increase in melanomas among younger, predominantly female individuals [55]. Other risk factors for low-CSD melanoma identified in case–control studies include the total number of moles, mole size, and clinically atypical or dysplastic moles [56]. The non-CSD group encompasses acral melanomas, certain melanomas in congenital nevi, melanomas in blue nevi, Spitz melanomas, mucosal melanomas, and uveal melanomas. Nodular and nevoid melanoma can occur through any pathway [53]. These classifications may contribute to enhanced therapeutic approaches.

The absence of marked solar elastosis was the most powerful morphologic criterion to predict the genotype of the BRAF^V600E^ mutation and is more reproducible among expert pathologists than the traditional designations of SSM, LMM, and NM [57,58].

## 4. Clinical Features

Melanomas can occur anywhere on the body, not just in sun-exposed areas. In New Zealand, the most common site in men is the back (~40% of melanomas), while in women, it is the leg (~35% of melanomas) (DermNet website). Although melanoma usually begins as a skin lesion, it can also develop on mucous membranes (mucosal melanoma), such as the lips or genitals. Occasionally, it occurs in other parts of the body, such as the eye, brain, mouth, or vagina.

The first sign of melanoma is usually an unusual-looking freckle or mole, which may itch or bleed. Melanomas may grow across the skin (radial growth phase) or in depth (vertical growth phase). Melanoma may be detected at an early stage when it is only a few millimeters in diameter, but it can grow to several centimeters before diagnosis. During its horizontal growth phase, melanoma is typically flat. As the vertical phase develops, the melanoma becomes thickened, raised, and palpable. Some melanomas are itchy or tender, while more advanced lesions may bleed easily or crust over. Most melanomas exhibit characteristics described by the ABCDE melanoma criteria [59,60].

White or pale skin color is an independent but significant risk factor for melanoma across diverse ethnic groups. However, individuals of all skin colors with a family history of melanoma are at increased risk due to genetic predisposition. In skin of color, melanomas can be harder to identify, as their growth phase and pattern may be masked or matched by the surrounding skin. People with skin of color tend to have thicker melanomas at diagnosis, higher mortality rates, and significantly higher rates of melanomas in non-sun-exposed areas, including subungual, palmar, and plantar surfaces, as well as non-cutaneous melanomas (NCCNs).

In the exploration of the intricate relationship between drug resistance and cellular death mechanisms, extensive research has demonstrated that melanoma cells possess the capability to evade apoptotic death by modulating pivotal proteins within the apoptotic pathway, thereby conferring resistance. Proteins belonging to the Bcl-2 family are notably implicated in this process, with elevated Bcl-2 expression serving to inhibit the liberation of cytochrome c from mitochondria and impede the downstream caspase cascade, thus impeding the initiation of the apoptotic program and leading to resistance to chemotherapeutic agents [61]. In the context of BRAF-mutant melanoma, the persistent activation of the MAPK signaling pathway not only augments cellular proliferation but also disrupts cell death signaling by upregulating the anti-apoptotic protein Mcl-1, ultimately resulting in resistance to BRAF inhibitors [62].

With respect to metastasis, considering programmed necrosis as an illustrative example, recent studies have revealed that dysregulation in the expression of necroptosis-associated proteins RIPK1 and RIPK3 triggers the generation of a cascade of pro-inflammatory signals within melanoma cells. These inflammatory cues are instrumental in reshaping the tumor microenvironment, thereby fostering the migration and invasion of tumor cells [63]. Furthermore, lipid peroxidation byproducts associated with ferroptosis have been implicated in inducing the secretion of matrix metalloproteinases (MMPs) by tumor cells to some extent. MMPs are capable of degrading the extracellular matrix, thereby facilitating the metastatic dissemination of melanoma cells [64].

## 5. The Cell Death Mechanisms of Current Systematic Therapy

Melanoma, a highly aggressive and metastatic skin cancer, presents significant therapeutic challenges due to tumor cell resistance and treatment-associated adverse effects. Different therapeutic strategies can activate distinct cell death pathways, offering promising avenues to optimize existing treatments and develop innovative approaches. Resistance to therapy in melanoma often arises from mechanisms that enable tumor cells to evade cell death, such as inhibiting apoptosis or activating survival signaling pathways. Understanding these mechanisms is critical for overcoming drug resistance and improving patient outcomes. By targeting these mechanisms in responsive patients, personalized therapeutic strategies can enhance efficacy while reducing adverse effects. Investigating cell death mechanisms in melanoma treatment is thus essential for advancing precision therapy and ultimately improving patient prognosis.

### 5.1. Apoptosis

**Figure 1:**
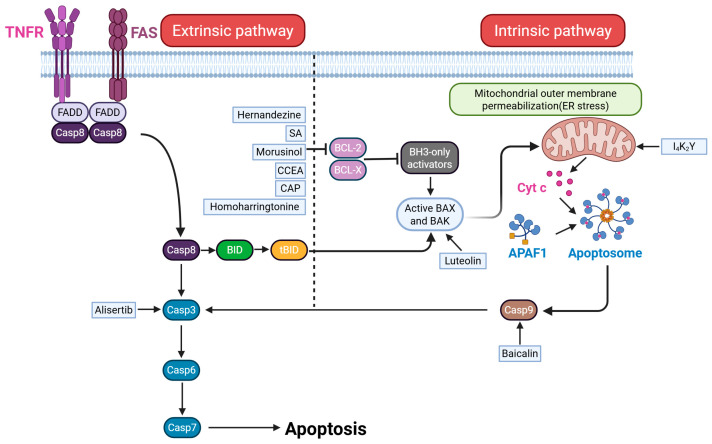
Apoptosis-related pathways in melanoma.

Apoptosis is an evolutionarily conserved form of programmed cell death that is essential for animal development and tissue homeostasis. Melanoma cells can undergo self-destruction via apoptosis [65] (Figure 1).

Alisertib (ALS), a selective inhibitor of Aurora kinase A (AURKA), has been employed in melanoma treatment [66]. ALS induces apoptosis and autophagy in A375 and skmel-5 cells by suppressing the p38 MAPK signaling pathway [66]. The therapeutic effect is synergistically enhanced when combined with a selective p38 MAPK inhibitor, as low levels of p38 MAPK promote ALS-induced apoptosis while reducing ALS-induced autophagy [66]. Recent research has revealed that certain phytochemicals contribute to melanoma apoptosis [67]. Hernandezine, an isolated compound from herbal medicine, inhibits proliferation and induces apoptosis in melanoma A375 and B16 cells [67]. Additionally, (S)-(-)-N-[2-(3-Hydroxy-2-oxo-2,3-dihydro-1H-indol-3-yl)-ethyl]-acetamide (SA), a novel compound derived from Selaginella pulvinata leaves, significantly inhibits melanoma cell proliferation and induces apoptosis and autophagy by potentially involving the PI3K/AKT and MAPK signaling pathways. Moreover, SA effectively retards melanoma tumor growth by reducing tumor volume and weight [68]. Morusinol derived from Morus alba induces cell cycle arrest and apoptosis by inhibiting the DNA damage response in melanoma through CHK1 degradation via the ubiquitin–proteasome pathway [69]. In addition, nanodrugs and new chemical drugs for melanoma also reveal significant anticancer effects based on apoptosis. Li et al. developed a novel redox and acid sensitive nanodrug (CDDP-CS-Cys-EA, CCEA) composed of chondroitin, antiangiogenic peptide, and cisplatin, which shows excellent antitumor activity and induces tumor cell apoptosis [70]. Amyloid-like aggregates of a short self-assembly peptide (Ac-IIIIKKDopa-NH2) block the exchange of biomolecules between the nucleus and cytoplasm, leading to cell apoptosis via S-phase arrest in the cell cycle and mitochondrial dysfunction [71]. Baicalin and its aglycon baicalein, major flavonoids derived from *Scutellaria baicalensis* Georgi, induce apoptosis and senescence in melanoma cells, suppressing tumorigenesis and tumor growth in vivo in melanoma models [72,73]. As a novel antitumor agent, starvation combined with atmospheric pressure plasma (CAP) leads to cell morphology changes, decreased metabolic activity, and reduced tumor burden in vivo [74]. Gracillin, Luteolin, and other compounds further enhance the overall impact, resulting in apoptosis [75,76]. Imatinib, a tyrosine kinase inhibitor, induces apoptosis and inhibits tumor cell proliferation in the presence of an activating KIT mutation. The response to imatinib in melanoma patients depends on the type of KIT mutation, with certain exon mutations correlating with better treatment responses [77]. However, due to the low incidence of KIT mutation-positive melanomas, imatinib’s use in melanoma therapy is limited [78]. These studies demonstrate that targeting melanoma cells through different treatment to induce apoptosis is an effective anticancer strategy in vitro and in vivo.

### 5.2. Anoikis

**Figure 2:**
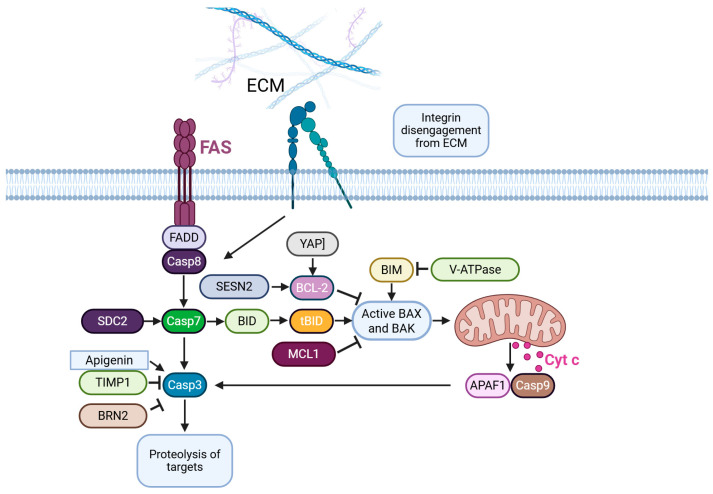
Anoikis-related pathways in melanoma.

Anoikis is a distinct form of apoptosis triggered by loss of contact with the extracellular matrix (ECM) [79], which is associated with vascularization and distant metastasis in cancer. Anoikis plays a central role in cancer metastasis. In melanoma, a variety of intracellular signaling cascades have been identified as potential drivers of anoikis resistance; however, a comprehensive understanding of the process remains elusive [80] (Figure 2).

He et al. identified several genes, including *FASLG*, *SOD2*, *BST2*, *PIK3R2*, *IKZF3*, *CDK2*, and *RAC3*, which are involved in anoikis [81]. The expression pattern of these hub genes related to anoikis may hold prognostic potential for cutaneous melanoma progression and overall patient survival. However, it is important to note that melanoma cells resistant to anoikis do not exhibit high sensitivity to chemotherapy. A study demonstrated that upregulation of syndecan-2 in melanoma cells enhances chemosensitivity through the activation of PI3K and ERK pathway-mediated anoikis suppression [82], suggesting a potential strategy for overcoming metastatic melanoma using chemotherapy. Apigenin, a nonmutagenic flavonoid compound, reduces integrin protein levels and inhibits phosphorylation of FAK and ERK1/2, thereby inducing anoikis in human cutaneous melanoma cells [83]. Another study reveals that simultaneous inhibition of Timp1 and AKT results in more effective suppression of anoikis in metastatic cells [84]. This finding provides new insights into the mechanisms by which Timp1 promotes cell survival in melanoma. Furthermore, other studies have uncovered an unreported role for STAT3 in mediating the upregulation of V-ATPase to promote resistance against anoikis [85]. Weems et al. reported that dynamic blebbing contributes to anoikis resistance in both NRAS- and BRAF-mutated melanomas, suggesting that the cell morphological program is a broadly adopted survival strategy in melanoma cells [86]. Zhao et al. demonstrated the YAP inhibitor CA3 significantly reduces anoikis resistance in melanoma cells via blocking YAP activity [87]. Low extracellular pH is also correlated with an anoikis-resistant phenotype [88]. Pierce and colleagues suggested that the induction of BRN2 expression alters signaling associated with resistance to anoikis [89]. Additionally, upregulated Sesn2 may facilitate ECM-detached melanoma cells in anoikis resistance, and Sesn2 mediates anoikis resistance by detoxifying intracellular ROS [90]. Adamo et al. reported that depletion of Mcl-1 renders melanoma cells susceptible to anoikis, and Mcl-1 protein turnover is regulated by the BRAF/MEK pathway [91]. Certain genes, such as SOCS-1, are associated with anoikis, and silencing SOCS-1 can inhibit subcutaneous tumor growth and metastatic development in the lungs in vivo [92]. These findings present an alternative approach for targeting cancer metastasis. In conclusion, understanding and harnessing the process of anoikis represents a significant avenue for combating melanoma.

### 5.3. Endoplasmic Reticulum Stress

**Figure 3:**
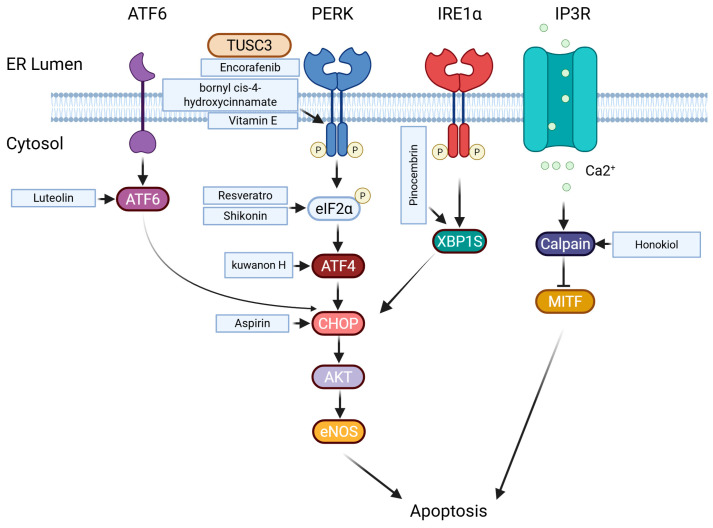
Endoplasmic reticulum stress-related death pathways in melanoma.

The handling, modification, and folding of proteins in the endoplasmic reticulum (ER) are tightly regulated processes that determine cell survival and death. Protein misfolding within the ER can have toxic consequences and is termed ER stress [93]. Abnormal changes in the tumor microenvironment can lead to alterations in ER homeostasis, promoting cancer progression [94]. Melanoma cells use various strategies to survive, like activating a response to unfolded proteins to resist ER stress-induced apoptosis. Oncogene activation halts this apoptosis, while increased ER chaperone proteins and BCL-2 family members enhance protein folding and raise the threshold for apoptosis [95] (Figure 3).

Hu et al. discovered a novel natural compound called kuwanon H (KuH), which induces cytotoxic ER stress, inhibits cell viability, and induces apoptosis [96]. Ausina et al. found that acetylsalicylic acid and salicylic acid trigger AKT/mTOR/AMPK-dependent activation of eNOS, increasing nitric oxide and reactive oxygen species production, which induces the ER stress response leading to the upregulation of the pro-apoptotic transcription factor CHOP expression [97]. Heo et al. investigated the effect of resveratrol on ER stress-induced apoptosis in the A375SM malignant melanoma cell line, demonstrating that resveratrol suppresses melanoma cell growth by stimulating ER stress through overexpression of elF2-α and CHOP [98]. Shikonin, a botanical drug extracted from Lithospermum erythrorhizon, triggers ER stress-mediated apoptosis by upregulating the expression of p-eIF2α, CHOP, and cleaved caspase-3 [99]. MiRNAs and traditional medicine also show obvious effects on ER stress. Grzywa et al. discovered that induction of ER stress-induced miR-410-3p upregulates AXL expression in vitro, leading to resistance to the BRAF inhibitor in melanoma [100]. Wang et al. investigated the functional significance of TUSC3, demonstrating its inhibitory effects on melanoma cells and its ability to induce ER stress through inhibition of the AKT/GSK3-β/β-catenin pathway, suggesting that TUSC3 could be a promising therapeutic target for melanoma treatment [101]. Chiu et al. used honokiol-induced ER stress CHOP activation to effectively suppress the growth and metastasis of melanoma by targeting MITF and β-catenin pathways simultaneously [102]. Zheng et al. evaluated pinocembrin, a natural flavanone with diverse biological and pharmacological activities, and discovered its ability to induce ER stress via the IRE1α/Xbp1 pathway in multiple melanoma cell lines [103]. Luteolin, a flavonoid compound, elicits apoptosis through elevating ROS-mediated ER stress [104]. Vemurafenib, a BRAF inhibitor, induces ER stress in BRAF-mutant melanoma when combined with RAF/MEK/ERK (MAPK) pathway inhibition, promoting cell death. This effect is also observed in NRAS-mutant melanoma [105]. Bornyl cis-4-hydroxy cinnamate activates the PERK and elF2α pathways, leading to the induction of ER stress-mediated apoptosis in melanoma cells [106]. Riaz Ahmed et al. discovered that 25-epi Ritterostatin GN1N induces cell death in melanoma cells through increased ER stress, highlighting its potential as an anticancer agent [107]. Montagnani Marelli et al. found that Vitamin E δ-tocotrienol (δ-TT) exerts its antitumor effects through activation of multiple branches related to ER stress-mediated apoptosis, including PERK/p-eIF2α/ATF4/CHOP, IRE1α, and caspase-4 pathways [108]. Activation of the ER stress-related pathways might represent an effective option for novel chemopreventive or therapeutic strategies for melanoma. Several chemocompounds have been identified as key contributors to ER stress. Mechanistic investigations have revealed that the novel Pyrrolidine Diketopiperazines 2155-14 and 2155-18 induce ER stress, which enhances basal autophagy and ultimately leads to melanoma cell death in BRAF- and NRAS-mutated melanoma cells. This effect is achieved through the binding of hnRNP H1 and H2 [109,110,111].

### 5.4. Autophagy

**Figure 4:**
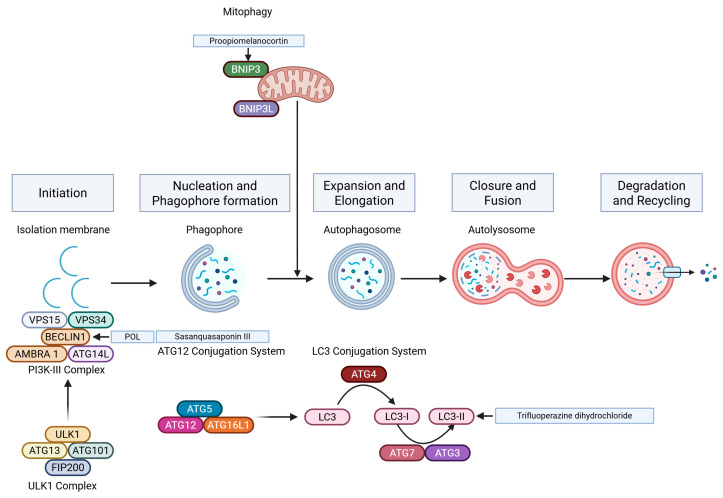
Autophagy-related death pathways in melanoma.

Autophagy is a eukaryotic cell degradation process that removes damaged or excess macrocomplexes and helps maintain cell metabolism and homeostasis. Reduced autophagy fuels melanoma formation, while increased autophagy sustains cell viability and drug resistance. Therefore, understanding autophagy regulation may advance melanoma treatment [112] (Figure 4).

Several studies have shown that inducing autophagy can inhibit the growth of melanoma cells [113,114]. Luan et al. found that Polygonatum odoratum lectin (POL) upregulates BECN1 expression by suppressing miR1290, promoting autophagic activity within cancerous cells and inducing cell death [115]. Additionally, Xiao et al. explored the involvement of miR-24-1-5p as an inducer of autophagy in melanoma cells, observing a significant increase in the LC3-II/I ratio and beclin-1 expression upon its activation, resulting in heightened cellular self-digestion and suppression of melanoma proliferation rates [116]. Proopiomelanocortin (POMC) has also been identified as a trigger for eliminating malignant melanocytes via autophagic processes [117]. Autophagy inhibition induced by TRPML1, a cationic channel localized in the lysosome, results in the accumulation of damaged mitochondria by blocking the mitophagic flux to lysosomes in melanoma cells [118]. The combination of doxycycline and minocycline increases autophagy levels and enhances anticancer effects in A375 and C32 cells compared to minocycline alone, providing a new drug idea for melanoma treatment [119]. As metastasis is a significant challenge in melanoma treatment, Xia et al. found that the antipsychotic drug trifluoperazine blocks the fusion of autophagosomes with lysosomes, leading to autophagosome accumulation and suppressing lung and bone metastasis [120]. Sasanquasaponin III, an important constituent of Theaceae, induces autophagy activation through the AKT/mTOR/p70S6K pathway and inhibits tumor metastasis in melanoma cells [121]. Liposomes containing paclitaxel and hydroxychloroquine also significantly inhibit metastatic melanoma via an autophagy-dependent pathway [122]. Therefore, targeting autophagy is emerging as a promising strategy for future cancer therapeutics.

### 5.5. Necroptosis

**Figure 5:**
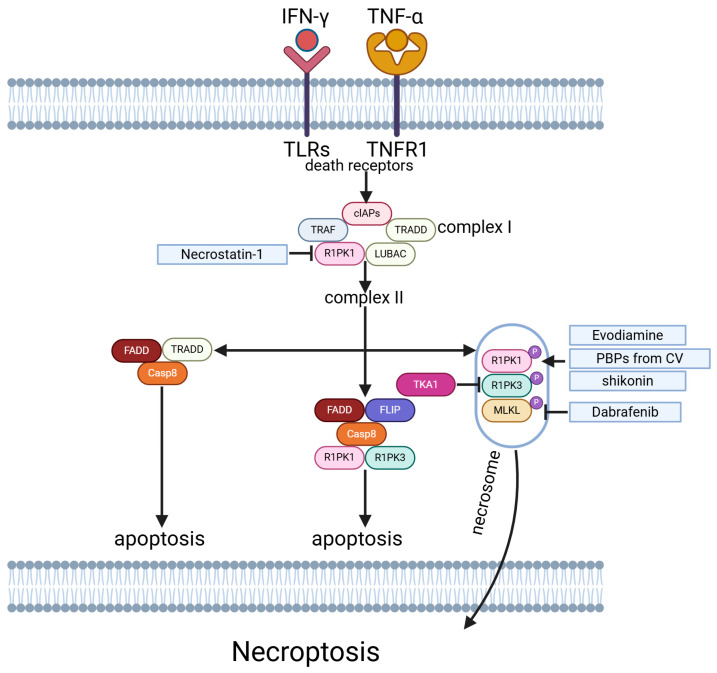
Necroptosis-related pathways in melanoma.

Necroptosis, alternatively termed regulated necrosis, is a mechanism of cellular demise contingent upon RIPK1 and RIPK3 kinases, occurring in a caspase-independent manner. Emerging as a novel category of programmed cell death, necroptosis exerts notable influences on tumor advancement and outcome [123]. In melanoma, necroptosis is a double-edged sword: it boosts immune response but also fuels tumor growth and metastasis [124,125] (Figure 5).

In the presence of a RIP3K mutation, the BRAF inhibitor Dabrafenib, unlike Vemurafenib, suppresses necroptosis in melanoma cells. Considering that RIPK3 expression can reveal necroptotic signaling, the reactivation of this pathway could hold therapeutic potential in the treatment of metastatic melanoma [126]. Basit et al. discovered that inhibition of mitochondrial complex I (CI) by BAY 87-2243 triggered an increase in mitophagy-dependent ROS, leading to necroptosis and ferroptosis-induced melanoma cell death in vivo [127]. Kong et al. found that a novel compound (10-Methoxy-1,2,3,4-tetrahydrobenzo(g) (1,3) diazepino(1,2-a) -(1,8) naphthyridin-6-yl) (phenyl) methanone (named 3u) could regulate the activity of caspase-8, which plays a crucial role in determining whether apoptosis or necroptosis occurs in melanoma cells [128]. Human and murine endothelial cells lacking TGF-β-activated kinase 1 (TAK1), a key factor in necroptosis, exhibit higher levels of necroptosis [129]. T Liu et al. developed a model based on necroptosis-related long non-coding RNAs (NRLs) for predicting the prognosis of patients with melanoma, which may provide a foundation for further investigation of melanoma necroptosis and offer new perspectives on clinical diagnosis and treatment approaches [130]. Evodiamine (EVO), a natural small molecule, exhibits anticancer effects. Liu et al. discovered that EVO induces apoptosis and necroptosis in human melanoma A375 cells through the generation of ROS [131]. A novel Pleuromutilin derivative (compound **38**), which increases cellular ROS levels, inhibited the growth of melanoma cancer cells in vivo through necroptosis [132]. Pawlikowska et al. found that protein-bound polysaccharides (PBPs) from Coriolus versicolor could induce RIPK1/RIPK3/MLKL-mediated necroptosis in amelanotic melanoma [133]. Shikonin, a Chinese herbal medicine, has been shown to induce necroptosis in melanoma cells through ROS production [134]. Additionally, compound **38**, a novel Pleuromutilin derivative that triggers the necroptosis pathway, has demonstrated low toxicity and excellent pharmacokinetic properties in animal models, making it a promising candidate for melanoma drug development [132]. Ilexgenin A can induce melanoma cell G1/S arrest in vitro and reduce tumor growth in vivo, accompanied by large areas of necrosis [135]. Homoharringtonine can induce melanoma cell G2/M arrest and lead to similar results [75]. These findings suggest that Chinese herbal medicine may have potential therapeutic applications for melanoma treatment, and triggering the necroptosis pathway may be a potential therapy strategy.

### 5.6. Pyroptosis

**Figure 6:**
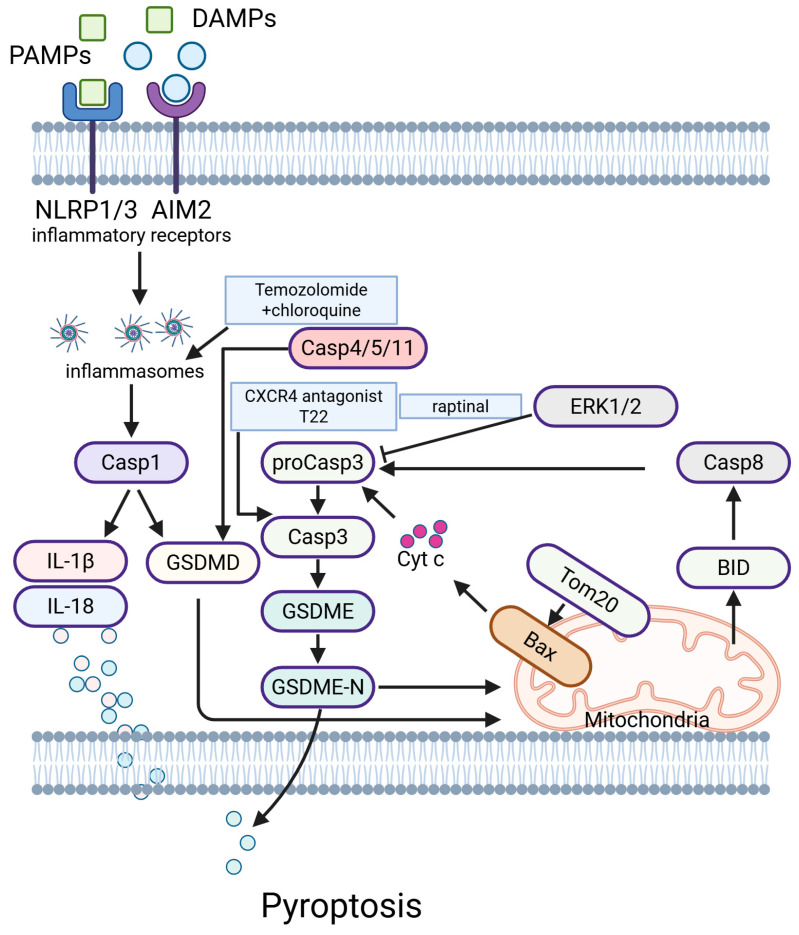
Pyroptosis-related pathways in melanoma.

Pyroptosis, a gasdermin-mediated programmed necrosis, can trigger the tumor inflammatory microenvironment and enhance the efficacy of tumor immunotherapy. Drug combinations performed in preclinical models based on conventional anticancer drugs demonstrate antitumor efficacy in melanoma via PYR induction. Moreover, several investigations have focused on the construction of PYR-associated gene signatures for predicting melanoma patient outcomes [136] (Figure 6).

Tom20, a component of the mitochondrial outer membrane complex, senses iron-activated ROS signaling to promote melanoma cell pyroptosis [137]. Zhao et al. reported a self-assembling nanotoxin (T22 peptide) that spatiotemporally targets CXCR4-expressing melanoma cells and initiates tunable cellular pyroptosis. The nanotoxin has a remarkable effect on antitumor efficacy and minimal side effects in melanoma in vivo [138]. Vernon et al. discovered that raptinal, a caspase-3 activator, induces pyroptosis in both human and mouse melanoma cell lines and delays tumor growth in vivo [139]. Ahmed and colleagues reported that exposure to a drug combination induced caspase-3 activation and GSDME/D cleavage, suggesting pyroptosis induction. Notably, the combination showed better antitumor activity in BRAFi-resistant CM143-bearing mice compared to temozolomide alone [140]. Zeng et al. used an experimental-based mechanistic investigation to demonstrate that exosomal miR-211-5p regulates pyroptosis and the immune microenvironment of melanoma through GNA15 [141]. Erkes and colleagues demonstrated that the antitumor effects of PLX4720 in combination with PD0325901 require a proficient immune system, and the combination of BRAFi/MEKi with drugs that stimulate pyroptosis represents a potentially life-saving treatment for melanoma [142]. Wang et al. showed that Phe-BF3 desilylation releases gasdermin from NP-GSDMA3 to induce pyroptosis [143]. These studies highlight the importance of pyroptosis in overcoming acquired resistance to targeted therapies in melanoma.

### 5.7. Ferroptosis

**Figure 7:**
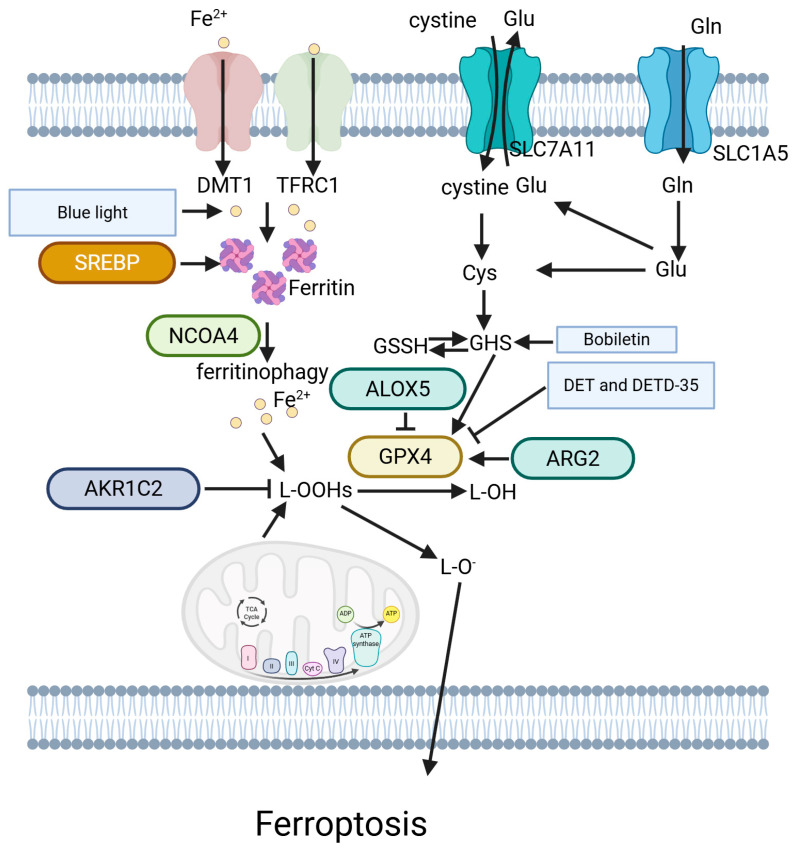
Ferroptosis-related pathways in melanoma.

Ferroptosis, an iron-dependent form of cell death, is characterized by overwhelming lipid peroxidation. Ferroptosis inducers have significant therapeutic potential for treating melanoma, acting as effective agents to overcome resistance to standard chemotherapy or targeted therapy. They can also be combined with targeted therapies or immune checkpoint inhibitors to enhance antitumor immunity and improve patient response rates [144] (Figure 7).

Several studies have identified genes that promote ferroptosis. In melanoma, BET inhibitor-induced AKR1C2 downregulation sensitizes ferroptosis [145]. MiRNAs are also involved in ferroptosis. Delivery of miR-21-3p sensitizes melanoma immunotherapy by activating ferroptosis [146]. Zhang et al. found that miR-9 reduces Erastin- and RSL3-induced ferroptosis by directly binding to the 3′-UTR region of GOT1 [147]. Similarly, Luo et al. discovered that miR-137 enhances the ferroptosis-mediated antitumor effects of Erastin by downregulating the glutamine transporter SLC1A5 [148].

Inhibiting GPX4 is a commonly employed strategy to induce ferroptosis. ARG2 suppresses ferroptosis by activating the AKT/GPX4 signaling pathway, reducing sorafenib-induced cell death in melanoma cells [149]. Wang et al. discovered that gambogenic acid (GNA) downregulates nuclear-enriched NEAT1, weakening the direct binding between SLC7A11 and NEAT1, further inhibiting GPX4 activity and subsequent ferroptosis through the AMPK/mTOR signaling axis-induced autophagy [150]. ALOX5 activates the AMPK/mTOR pathway and inhibits GPX4 expression, promoting autophagy-dependent ferroptosis [151]. MitoCur-1, a curcumin derivative, significantly induces the inhibition of USP14 and the inactivation of the GPX4 enzyme by decreasing SLC7A11 expression [152]. Phyto-sesquiterpene lactones DET and DETD-35 induce ferroptosis through metabolic reprogramming in a GPX4 inhibition manner [153]. Li et al. found that activation of TRPV4, a calcium-permeable TRP ion channel, facilitates ferroptosis in melanoma cells [154], providing insight into how other signals participate in ferroptotic processes.

Interestingly, light also contributes to ferroptosis. Yang et al. discovered that blue light induces the upregulation of SVCT2 expression, resulting in elevated intracellular Vitamin C concentration through the NF-κB signaling pathway. The increased cellular Vitamin C, along with Fe^2+^ generated by blue light exposure, triggers melanoma ferroptosis [155]. When combined with low-level laser, gallic acid (GA), a natural polyhydroxy phenolic compound commonly found in foods, contributes to melanoma ferroptosis [156]. Vadarevu et al. demonstrated that light-activated protoporphyrin IX-based polysilsesquioxane nanoparticles (PpIX-PSilQ NPs) also induce ferroptosis in melanoma cells [157]. 

Additionally, numerous other treatments show promising development prospects based on ferroptosis. Nobiletin, a natural product isolated from citrus peel, induces ferroptosis by modulating the GSK3β-mediated KEAP1/NRF2/HO-1 signaling pathway in human melanoma cells [158]. Some regulators exert suppressive effects on ferroptosis. The lipogenesis regulator SREBP2 directly promotes the transcription of transferrin (TF), an iron carrier protein that reduces intracellular iron pools, conferring resistance against ferroptosis [159]. These studies show that ferroptosis could offer new treatment options for melanoma.

### 5.8. Cuproptosis

**Figure 8:**
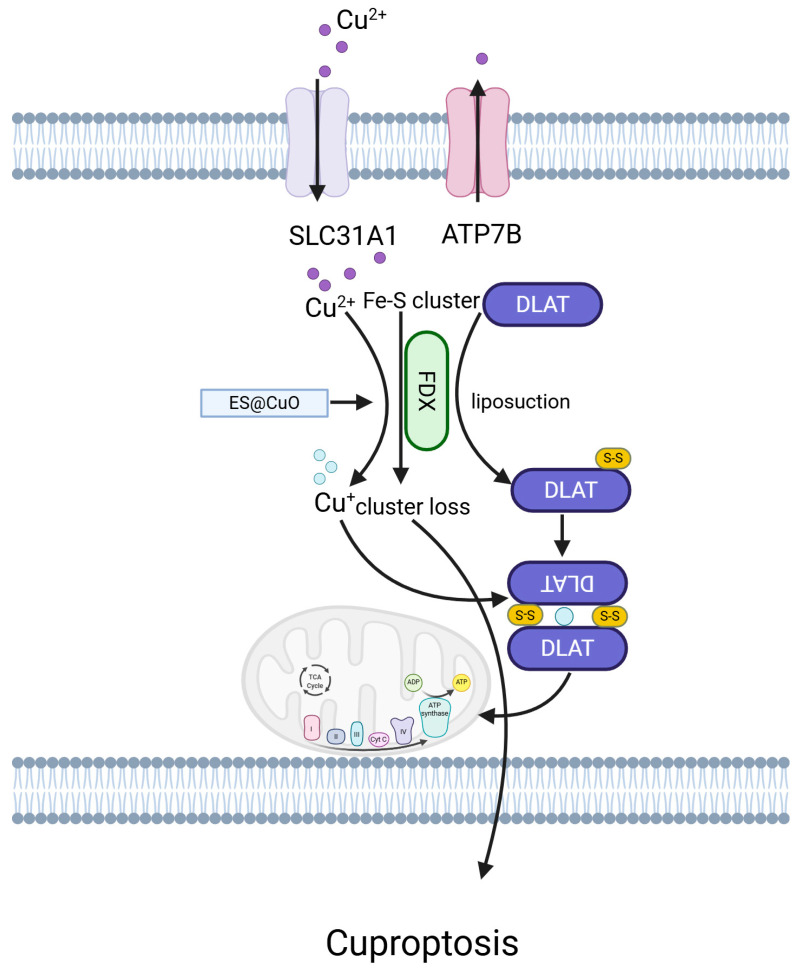
Cuproptosis-related pathways in melanoma.

In recent years, cuproptosis, a novel copper-dependent form of regulated cell death, has been identified. Cuproptosis is triggered by copper binding to lipoylated enzymes in the tricarboxylic acid (TCA) cycle, leading to protein aggregation, proteotoxic stress, and ultimately, cell death [160]. Exploring the link between molecular subtypes of cuproptosis-related genes (CRGs) and melanoma metastasis could help in melanoma prognosis pre dictions [161] (Figure 8).

These pathways are activated in response to copper-induced stress, leading to phosphorylation cascades that modulate the expression of pro-apoptotic genes and enhance cell death [162]. Additionally, cuproptosis is characterized by the production of reactive oxygen species (ROS) and the alteration of mitochondrial function, further contributing to the cell death process [163]. The role of cuproptosis in diseases such as cancer has gained significant attention. Studies have shown that copper-chelating agents can inhibit cuproptosis, suggesting potential therapeutic strategies for targeting this form of cell death in cancer treatment [164]. Furthermore, understanding the molecular mechanisms underlying cuproptosis may lead to the development of novel biomarkers and therapeutic agents for the early detection and treatment of melanoma and other cancers [165]. In conclusion, cuproptosis represents a fascinating area of research with important implications for our understanding of cell death mechanisms and their role in disease. As research progresses, we anticipate that cuproptosis will continue to provide new insights into the complex interplay between copper metabolism, cell death, and human health. The different manners of cell death and corresponding mechanisms are shown below (Table 2).

## 6. Current Clinical Treatment for Melanoma

Immunotherapy and targeted therapy have revolutionized the treatment landscape of melanoma. Currently, more than 500 clinical trials are actively progressing worldwide, aiming to discover novel treatment approaches for patients (data source: ClinicalTrials.gov). New molecular therapies, including cell therapy, therapeutic vaccines, and antibody–drug conjugates (ADCs), have taken up a significant share in the research and development pipeline. Furthermore, the exploration of combination therapies represents a pivotal area of focus within the oncology industry.

To date, seven immune checkpoint inhibitors have been approved by global regulatory agencies for the treatment of melanoma, targeting various checkpoint proteins such as PD-1, PD-L1, and LAG-3 [164]. A number of registered clinical trials are presently assessing pharmaceutical agents for melanoma treatment, encompassing targeted and immunotherapeutic approaches. Preclinical research has pinpointed several potential targets for future melanoma therapies, such as CD126, chondroitin sulfate proteoglycan 4 (CSPG4), the combination of CD70 and B7-H3, and αvβ3 integrin [165]. Furthermore, innovative pharmacologic approaches are being examined, including oncolytic virus therapy and methods to enhance the efficacy of immunotherapy. Immunotherapy has indeed transformed the landscape of melanoma treatment, offering promising results for previously resistant forms of the disease. However, its effectiveness varies significantly among patients, posing a limitation. Due to largely unknown factors, only approximately 50% of melanoma patients achieve long-term survival with immunotherapy [166]. Consequently, while researchers are exploring novel pharmacologic agents, a significant portion of current pharmacologic research in melanoma is directed towards identifying predictors of immunotherapy success and developing strategies to enhance its efficacy in refractory cases.

For instance, the composition of the gastrointestinal microbiome has garnered recent attention as a predictive biomarker for the outcomes of immunotherapy [167]. Consequently, recent research has explored microbiota-modulating interventions, such as fecal microbiota transplantation, in conjunction with immunotherapy among patients with advanced melanoma. In 2021, clinical responses were observed in three out of ten patients who had previously been resistant to anti-PD-1 treatment, following fecal microbiota transplantation and the reinstatement of immunotherapy [168]. Likewise, a study revealed clinical benefits in six out of fifteen previously resistant patients who underwent fecal microbiota transplantation in combination with anti-PD-1 immunotherapy [169]. Currently, additional clinical trials are underway to evaluate the use of fecal microbiota transplantation in patients with advanced melanoma (NCT03893019, NCT04577729, NCT05251389, and NCT04988841).

Targeted therapy represents a pivotal direction in the development of novel pharmaceuticals for metastatic melanoma. Currently, three BRAF inhibitors, namely Vemurafenib, Dabrafenib, and Encorafenib, are in clinical use [170]. These agents selectively target cell lines harboring the BRAF^V600^ mutation, effectively inducing tumor cell death. Their efficacy in halting cell growth is remarkable, achieved by suppressing the MAPK signaling pathways through the reduction of MEK and ERK phosphorylation [171]. MEK inhibitors have been developed and initially approved for the treatment of BRAF-mutated metastatic melanoma in patients naive to BRAF inhibitors, aiming to prevent the overactivation of the MAPK pathway in melanoma cells [16]. Notably, the combination of Trametinib, a MEK inhibitor, with Dabrafenib, a specific BRAF^V600E/K^ inhibitor, has gained FDA approval for the treatment of various solid tumors, including melanoma, between 2014 and 2023 [172]. Additionally, two other MEK inhibitors, Cotellic and Mektovi, have been approved for melanoma treatment, either as monotherapy or in combination with other MAPK inhibitors [173]. Current research emphasizes the RAS/RAF/MAPK pathway as a crucial therapeutic target, particularly in the context of precision medicine, where combination therapies are being explored. A CRISPR/cas9 study, for instance, demonstrated that the deletion of KEAP1, in conjunction with specific RAS/RAF/MAPK pathway inhibitors, alters cell metabolism, enabling proliferation independently of MAPK signaling [174]. Furthermore, BRAF^V600^ mutations are prevalent in the majority of acquired melanocytic nevi, accounting for over 80% of cases [175]. Other somatic mutations in melanoma, such as those affecting the tyrosine kinase receptor c-KIT (3%) or the NRAS protein (15–25%) [176,177], primarily lead to the overactivation of the MAPK pathway. Rarer mutations involve molecules regulating cell growth and survival through alternative intracellular pathways, like the AKT/PKB pathway [178]. However, specific drugs targeting these mutations are currently unavailable.

Cell and gene therapies (CGT) have seen an increase in clinical interventional studies initiated, completed, or currently active since 2019. These studies include clinical trials for a broader range of solid tumors, such as melanoma [179,180]. The purpose of most current clinical trial drugs is to inhibit the proliferation of cancer cells, but the specific mode of cell death they induce is not entirely clear. Therefore, if the specific mechanism of drug-induced cell death can be clarified, and activators/inhibitors of that mode of death are used in combination, it may be possible to more effectively achieve the goal of treating melanoma.

Despite advancements in pharmacological therapies for advanced melanoma, challenges remain in evaluating and comparing these agents. Studies often include patients with unique genetic or phenotypic characteristics, or those resistant/untreated with prior regimens. Including patients at various disease stages yields incomparable objective response rates. Trials vary in long-term follow-up duration, potentially missing long-term safety and resistance data. Cost-effectiveness studies are essential due to medication cost and availability differences.

## 7. Discussion

Malignant melanoma, a potentially preventable neoplasm, predominantly impacts the integumentary system, ranking it among the most common malignancies. To formulate highly effective therapeutic strategies for melanoma, a thorough comprehension of its underlying mechanisms and progression patterns is of utmost importance. In the early phases, complete surgical excision usually leads to favorable outcomes, with a relatively high survival rate. Nevertheless, once melanoma metastasizes, the survival time of patients is markedly shortened [181,182]. According to the SEER (Surveillance, Epidemiology, and End Results Program), the most recent 5-year survival rate (2013–2019) for melanoma in the United States is 93.5%, while the survival rate for stage IV disease is merely 29.8% [183] (data source: US Mortality Files, National Center for Health Statistics, CDC. National Cancer Institute Melanoma of the Skin—Cancer Stat Facts. Available online). Undoubtedly, with the advancement of new preventive strategies, screening techniques, accurate diagnostic methods, and treatment modalities, the 5-year overall survival rate for melanoma patients is steadily increasing [184].

Despite the availability of a wide range of treatment options, managing melanoma still poses significant challenges. The drug resistance exhibited by BRAF mutant patients treated with BRAFi + MEKi and those undergoing immunotherapy is a formidable obstacle in melanoma management [185]. Moreover, metastasis and toxic side effects continue to be major impediments. For example, the activation of YAP in melanoma promotes resistance to anoikis and metastasis, highlighting the complex nature of this disease [87]. Metastatic melanoma represents a substantial clinical challenge, as it is the primary cause of melanoma-related mortality. The current therapeutic landscape faces difficulties in offering curative solutions for advanced stage melanoma. Novel approaches are urgently needed to address the complexities of metastasis and develop treatments that can effectively target disseminated melanoma cells.

Several complex mechanisms, such as necroptosis (a form of programmed cell death), anoikis (a mechanism that inhibits the survival of detached cells), ferroptosis (an iron-dependent form of cell death), and autophagy (a cellular recycling process), play pivotal roles in regulating melanoma progression. Comprehending and manipulating these mechanisms is fundamental for the development of targeted therapies against melanoma. In the quest for effective treatments, researchers are delving into substances derived from herbal medicine and plants [186,187,188,189]. Nature has demonstrated its richness as a source of bioactive compounds with potential anticancer properties. These substances may exert their effects through diverse mechanisms, making them promising candidates for the development of drugs that can combat melanoma from multiple perspectives. The synergy between these natural compounds and modern medical approaches offers a promising path for improving the outcomes of melanoma treatment.

In medical research, the study of melanoma resistance and metastasis is crucial. These characteristics are the main reasons for treatment failure and poor prognosis. In-depth research into these issues is significant for improving patient survival rates and quality of life. Aged fibroblasts secrete sFRP2, which acts as a Wnt antagonist. This secretion sets off a multi-step signaling cascade within melanoma cells. As a result, the levels of β-catenin and MITF decline, ultimately culminating in the loss of APE1, a key redox effector. The absence of APE1 weakens the melanoma cells’ response to DNA damage triggered by reactive oxygen species, thereby increasing their resistance to targeted therapy, such as vemurafenib. Moreover, the age-related rise in sFRP2 also bolsters angiogenesis and metastasis in melanoma cells [190]. MAPK-targeted therapy elicits similar signatures in melanoma, signifying a non-genomic form of resistance to anti-PD-1 therapy. Validating IPRES in other tumor cohorts has identified a transcriptomic subset across diverse advanced cancers. These findings suggest that zeroing in on the biological processes underlying IPRES could potentially enhance the anti-PD-1 response in melanoma and other cancer types [191]. Treatment with RAF inhibitors gives rise to a diverse range of clinical genetic resistance mechanisms, mainly revolving around the reactivation of the MAPK pathway [192]. All cell death processes share common signaling pathways, including mitochondrial failure and the TNF signaling pathway. In necroptosis, mitochondrial dysfunction drives cell death via the RIPK1/RIPK3/MLKL pathway [193]. Combining BH3 analogs with BRAF/MEK inhibitors can boost apoptosis in melanoma cells [194]. In several preclinical studies, metformin has demonstrated antitumor activity against melanoma, especially in cases with BRAF mutations [195].

Mutations in the oncogenes BRAF and MEK are commonly observed in melanoma, leading to the hyperactivation of the MAPK signaling cascade. This cascade plays a pivotal role in promoting the proliferation and survival of tumor cells. Therapeutics, specifically BRAF inhibitors and MEK inhibitors, are developed with the intention of disrupting this pathway. However, tumor cells demonstrate a remarkable ability to evade apoptosis through various mechanisms, including the upregulation of anti-apoptotic proteins within the Bcl-2 family and the downregulation of pro-apoptotic proteins, such as BAX and BAK. As a result, these cells are capable of resisting apoptotic signals and continuing to proliferate even when the BRAF and MEK pathways are inhibited [196]. In the realm of immunotherapy, the immune system strives to recognize and eliminate tumor cells. Nevertheless, tumor cells possess the ability to evade apoptosis, allowing them to survive despite immune attack. They may achieve this by downregulating the Fas ligand or upregulating Fas antagonists, thereby impeding the immune system’s capacity to induce tumor cell apoptosis through the Fas-FasL pathway [197]. This mechanism plays a significant role in the development of resistance to immunotherapy.

Ultraviolet radiation (UVR) can induce DNA damage, which in turn triggers gene mutations that drive the development of melanoma, UVR also promotes the proliferation and survival of melanocytes by activating carcinogenic signaling pathways, while suppressing immune responses, thereby facilitating tumor evasion and progression. On the other hand, UVR can also induce apoptosis in melanoma cells and activate immune responses, which may help control tumor growth. The impact of UVR on melanoma is dose-dependent: low doses may enhance cell survival, whereas high doses can trigger cell death. Moreover, UVR affects the production of melanin, having both protective and cytotoxic effects on melanoma cells. Understanding these two effects of UVR is crucial for developing effective strategies for the prevention and treatment of melanoma [198,199]. The vitamin D signaling pathway plays a critical role in tumor suppression through interactions with its active metabolites, such as 1,25-dihydroxyvitamin D3, which bind to the vitamin D receptor (VDR) and other nuclear receptors like RORα and RORγ. This binding inhibits cancer cell proliferation, promotes differentiation, and enhances immune responses. However, abnormalities in this pathway, such as reduced VDR expression, are closely linked to melanoma progression and poor prognosis. Recent discoveries in the CYP11A1-dependent vitamin D metabolic pathway offer promising new therapeutic targets for melanoma prevention and treatment. Future research directions include leveraging bioinformatics and artificial intelligence to analyze multi-omics data for melanoma, identifying novel therapeutic targets and biomarkers. Additionally, exploring synergistic effects between the vitamin D signaling pathway and other treatment strategies, such as immunotherapy and targeted therapy, is a priority. Developing personalized treatment regimens tailored to melanoma heterogeneity and drug resistance remains a critical focus [200].

Melanoma, originating from melanocytes, exhibits treatment sensitivity to immunotherapy, chemotherapy, and radiotherapy influenced by melanin production. The intricate interplay between melanin and the tumor microenvironment significantly impacts melanoma progression. Elucidating the dual role of melanin in melanoma development and treatment is essential for optimizing therapeutic strategies and enhancing patient prognosis [37]. Melanin facilitates tumor cell immune evasion and disrupts host homeostasis by secreting factors such as CRH, urocortin, POMC derivatives (ACTH, MSH, endorphins), and biogenic amines (catecholamines, serotonin, melatonin), which regulate the HPA axis, sympathetic nervous system, cortisol levels, immune responses, and metabolism. These mechanisms contribute to an environment conducive to tumor growth, accelerating melanoma progression and compromising host health [201]. POMC and its derivatives exert multifaceted effects on melanoma progression and systemic physiology. At the cellular level, POMC derivatives such as α-MSH and ACTH enhance melanin synthesis by activating MC1R on melanocytes, increasing cAMP levels, tyrosinase activity, and melanin production. They also regulate melanoma cell proliferation, differentiation, migration, and invasion through cAMP-dependent signaling pathways and the modulation of cytoskeletal reorganization. Systemically, POMC derivatives influence skin pigmentation, immune function, endocrine responses, and nervous system activity, with significant implications for melanoma development, metastasis, and systemic responses [35,36].

Autophagy exhibits a dual role in tumor cells. Firstly, it maintains cellular homeostasis by degrading damaged organelles and proteins, thereby facilitating the survival of tumor cells under stressful conditions. When tumor cells are treated with BRAF inhibitors and MEK inhibitors, they may enhance autophagic flux to eliminate the damaged organelles and abnormal proteins induced by these agents. This adaptive response enables them to manage the stress imposed by the drugs, ultimately contributing to drug resistance [202]. Secondly, autophagy can negatively regulate cell proliferation signaling pathways by degrading crucial signaling molecules. However, in certain instances, tumor cells may disrupt the normal process of autophagic flux, preventing it from effectively inhibiting proliferation signals. This alteration plays a crucial role in the development of resistance to BRAF inhibitors and MEK inhibitors [203]. Currently, researchers are actively exploring the combined application of autophagy inhibitors (e.g., chloroquine) with apoptosis inducers [204]. The mechanism of action of autophagy inhibitors is to block the pathways by which tumor cells utilize autophagy to maintain their survival. Taking chloroquine (CQ) and hydroxychloroquine (HCQ) as examples, these autophagy inhibitors can prevent the fusion of autophagosomes with lysosomes, thereby interrupting the autophagy process. Preclinical studies indicate that combining CQ or HCQ with BRAF inhibitors, MEK inhibitors, or immunotherapy can enhance the cytotoxic effects of these drugs on melanoma cells [205,206,207,208,209] (NCT01844505). In certain cases, moderate activation of autophagy may also improve the responsiveness of tumor cells to treatment. For example, certain natural compounds, such as rapamycin, can activate autophagy and enhance treatment effects by inducing autophagic cell death in tumor cells [210]. However, it is necessary to carefully control the degree and timing of autophagy activation to prevent excessive activation of autophagy from enhancing the survival ability of tumor cells.

The tumor microenvironment (TME) exerts a complex and crucial influence on the death of melanoma cells. Comprising various cellular and non-cellular components, such as immune cells, fibroblasts, vascular endothelial cells, the extracellular matrix (ECM), and signaling molecules, the TME impacts the survival, proliferation, and death of melanoma cells through intricate interactions. Cytotoxic T lymphocytes (CTLs) have the ability to recognize and eliminate melanoma cells. However, regulatory T cells (Tregs) and myeloid-derived suppressor cells (MDSCs) within the TME counteract this effect, facilitating the survival of tumor cells [211]. IFN-γ and TNF-α can induce apoptosis in melanoma cells [212]. Under hypoxic conditions, the activation of HIF enables melanoma cells to adapt to a low-oxygen environment, augmenting their survival capabilities, yet it can also trigger cell apoptosis or necrosis [213]. Collagen and fibronectin affect the survival and death of melanoma cells through the integrin signaling pathway [214]. In summary, the TME impacts the death of melanoma cells through multiple mechanisms, which can either promote or inhibit cell death. Comprehending these mechanisms can aid in the development of more effective treatment strategies, such as immunotherapy, targeted therapy, and combination therapy, to enhance the treatment outcomes of melanoma.

In the field of melanoma treatment, the interaction between apoptotic and non-apoptotic cell death mechanisms constitutes a key research area, as it is directly related to treatment efficacy and resistance issues. The combined induction of apoptosis (such as through the use of BH3 mimetics) and necroptosis (such as through the use of RIPK1 inhibitors) has been shown to significantly enhance the mortality rate of melanoma cells [215]. Additionally, drugs that induce ferroptosis (like Erastin or sorafenib) can synergize with apoptosis inducers [216]. Researchers are developing or seeking drugs that can specifically induce apoptosis in melanoma cells and exploring their combined use with BRAF inhibitors, MEK inhibitors, or immunotherapy. For example, certain small molecule compounds can directly activate pro-apoptotic proteins, such as BH3 mimetics, which release pro-apoptotic proteins by binding to anti-apoptotic members of the Bcl-2 family, thereby inducing cell apoptosis. Clinical studies indicate that combining BH3 mimetics with existing melanoma treatment drugs may increase the sensitivity of tumor cells to treatment. At the same time, in-depth research into key nodes in apoptosis-related signaling pathways and the development of inhibitors or activators targeting these nodes are also important research directions. For instance, the PI3K/AKT/mTOR signaling pathway is closely related to apoptosis, and inhibiting this pathway can enhance the sensitivity of tumor cells to apoptosis [217]. Therefore, the combined use of PI3K inhibitors or mTOR inhibitors with existing treatment regimens can be considered to overcome treatment resistance caused by apoptosis evasion.

In clinical practice, the targeting of cell death pathways is fraught with limitations and adverse effects. The specificity of apoptosis-inducing agents proves inadequate, posing potential toxicity to normal cellular structures. As an illustration, specific BH3 mimetics exhibit the capacity to elicit apoptosis in melanoma cells; however, their toxicity towards normal tissues imposes restrictions on dosage administration, thereby undermining treatment efficacy [218]. The mechanisms underlying tumor cell evasion of apoptosis are intricate, rendering it challenging for a solitary inducer to counteract these mechanisms. While autophagy inhibitors have been shown to augment therapeutic outcomes, excessive inhibition may result in the accumulation of cytotoxic compounds, ultimately fostering tumor cell viability. The precise modulation of the timing and dosage of autophagy activators remains elusive, and the clinical utility is further hampered by the absence of reliable predictive biomarkers. The development of necroptosis-targeted therapeutics is still in nascent stages, with the potential for targeting interfering with normal physiological functions [219,220]. Additionally, individual variations and the absence of effective screening methodologies restrict their clinical deployment.

The adverse effects associated with apoptosis-targeted therapies primarily encompass bone marrow suppression, which results in a decline in hematological indices, particularly leukocytes and platelets [221]. This decrement subsequently elevates the susceptibility to infections and hemorrhages. During melanoma therapy, some patients experience a decrease in leukocyte levels after receiving apoptosis-inducing drugs. Additionally, this treatment strategy can also cause gastrointestinal problems, such as nausea, vomiting, and diarrhea, which may affect the patients’ quality of life and their adherence to treatment schedules [222]. Moreover, autophagy inhibitors have the potential to disrupt the metabolic processes and homeostatic mechanisms of normal cellular function. Prolonged usage of chloroquine, for instance, can induce retinopathy, ultimately compromising visual acuity [223]. Conversely, autophagy activators, such as rapamycin, may elicit immunosuppressive effects, thus augmenting the risk of infections [224]. The employment of necroptosis agonists leads to an elevation in body temperature and marked increases in serum levels of inflammatory cytokines, including IL-6 and TNF-α [225,226]. Consistently, clinical trials have revealed similar inflammatory manifestations in some patients, which adversely impact drug tolerance and treatment safety profiles [227,228,229] (NCT01909453, NCT01006980, and NCT01909453).

Recent NT (nanotechnology) breakthroughs highlight its key role in medical fields, especially oncology [230]. Nanomedicine, a part of NT, offers new ways to treat various diseases, including melanoma. NPs (nanoparticles) have unique features like a small size and large surface area, which improve drug delivery and treatment results [231]. Precision in nanomedicine is crucial for melanoma treatment. Embedding drugs in NPs allows for the accurate targeting of melanoma cells, reducing damage to healthy tissues [232,233,234]. This method shows promise in overcoming traditional treatment limits like toxicity and poor drug penetration. NT progress, combined with natural compound research, paves the way for multi-mechanism melanoma therapies. The integration of nanomedicine with traditional and new treatments gives hope to melanoma patients. Collaboration among scientists, clinicians, and industry is essential for better melanoma treatment.

The response of melanoma cells to treatment often exhibits heterogeneity, which implies that not all melanoma cells will react to a specific treatment in an identical manner. There are seven subtypes of melanoma. SSM is the most common subtype in Western countries. In SSM, the BRAF mutation is the most common. Less commonly, CCDN1 is found in about 6% of SSM, and PTEN and TP53 are observed in more advanced tumors [46]. In this subtype, BRAF^V600E/K^ inhibitors, MEK1/2 inhibitors, or a combination could be considered. NM is the second most common type of melanoma, comprising 14–15% of all cutaneous melanoma cases [235]. Besides the BRAF mutation, NM is more frequently associated with upregulated PD-L1 and NRAS mutation. Immune-checkpoint inhibition therapy has been attempted for this type [236]. LMM is the third most common subtype. Mutations with LMM include BRAF, CCDN1, NRAS, TERT, KIT, PTEN, CDKN2A, and TP53 [46]. ALM often occurs on the hands and feet. Mutations affecting the ALM include CCDN1, CDK4, C-KIT, NRAS, PTEN, NTRK, BRAF, ALK, and CDKN2A [46]. BRAF and MEK inhibition combinations have proven to work well in BRAF-mutated melanomas, but they have limited efficacy in this subtype [237]. Meanwhile, inhibitors against PI3K/AKT/mTOR, CDK, and MDM2/p53 are currently being explored as potential treatment options [238]. Accounting for less than 4% of melanomas, DM is a less frequent subtype. The PD-L1 inhibitor has been demonstrated to be useful for neoadjuvant use in DM with 70% of patients having an objective tumor response and 32% showing a complete response [239]. Nevoid melanoma is rare, and makes up no more than 0.5% of all melanoma. Up to now, no data exist on genetic mutations of this type.

In summary, the development of personalized treatment plans assumes paramount importance [240,241]. By acquiring a more profound understanding of the tumor characteristics of diverse patient populations, encompassing their unique gene expression profiles, the activity of cell death pathways, and potential resistance mechanisms, physicians can furnish patients with more tailored and efficacious treatment strategies. Furthermore, the incessant progression of nanotechnology and natural compound research has augmented the prospects of multi-mechanism combination therapy for melanoma. These nascent treatment modalities are anticipated to impart fresh hope to patients and substantially enhance the therapeutic outcomes for melanoma, thereby ensuring a more rigorous and academic approach to the management of this disease.

## 8. Conclusions

In general, the multifaceted nature of melanoma necessitates a comprehensive and integrative approach to treatments. As we delve deeper into the intricacies of melanoma biology, the future holds promise for more efficacious therapies. Continued investigation of molecular and cellular processes driving melanoma progression will undoubtedly reveal novel therapeutic targets. In-depth study of the precise mechanisms of drug-induced cell death in clinical trials, and the use of corresponding activators or inhibitors, may more effectively achieve the goal of treating melanoma.

## Figures and Tables

**Table 1:** Summary of several gene mutations in melanoma.

Gene Mutations	Mechanism
*NARS/BRAF*	Activates the RAS/RAF/MAPK/ERK signaling pathway [25,38]
*KIT*	Leads to constitutive activation of c-KIT tyrosine kinase activity and subsequent induction of both MAPK and PI3K/AKT pathways [27,39]
*NF1*	Negative feedback on RAS, thereby causing hyperactivation of the MAPK and PI3K/mTOR signaling pathways [40]
*CDKN2A*	Impairs two of the most important tumor suppressor pathways, Rb and p53 [41,42]
*CDK4*	Leads to the amino acid substitution and prevents the binding of p16 to the catalytic subunit in the Rb pathway, triggering constitutive activation of the CDK4 kinase [43]
*POT1*	Impairs the function of shelterin complex [44] and confers a telomere instability phenotype [45]
*TERT*	Activates the MAPK pathway [46] and increases telomerase activity resulting in chromosomal instability [47]
*MITF*	Regulates multiple biological processes in melanoma cells such as differentiation, proliferation, migration, and senescence by activating several pathways such as the BRAF^V600E^/ERK1/2 and Wnt/β-catenin pathways [48]
*MC1R*	Impairs the cAMP pathway mediated by ligand–receptor interaction and increases risk for melanoma secondary to intensified UV-mediated DNA damage in the setting of absent photoprotective eumelanin [49,50]
*PTEN*	Activates the PI3K/AKT/PTEN pathway [51]

**Table 2:** Potential targeted drugs for different cell death mechanisms in melanoma.

Manner of Death	Regulated Pathways of Cell Death	Corresponding Treatment
Apoptosis	Inhibition of the p38/MAPK pathway	Alisertib (ALS) [66]
	Inhibition of the PI3K/AKT and activation of MAPK pathway	(S)-(-)-N-[2-(3-Hydroxy-2-oxo-2,3-dihydro-1H-indol-3-yl)-ethyl]-acetamide (SA) [68]
	Activation of the AMPK–mTOR pathway	Hernandezine [67]
	Inhibiting DNA damage response through CHK1 degradation via the ubiquitin–proteasome pathway	Morusinol [69]
	Upregulation of Bax and downregulation of Bcl-2	CCEA (composed of chondroitin, antiangiogenic peptide, and cisplatin) [70]
	Blocking the exchange of biomolecules between the nucleus and cytoplasm	Ac-IIIIKKDopa-NH2 [71]
Anoikis	Inhibition of the FAK/ERK1/2 pathways	Apigenin [83]
	Inhibition of the Timp1-mediated PI3K/PDK1/PKC pathway	shTimp1/AKT-siRNA [84]
	Inhibition of YAP activity	CA3 [87]
	Inhibition of c-MET by targeting BRN2	Foretinib or capmatinib [89]
	Increasing the cellular level of ROS	shSesn2 [90]
	Inhibition of the Mcl-1-mediated BRAF/MEK pathway	BH3 mimetic targeting Mcl-1 [91]
Endoplasmic reticulum stress	Regulation of the ATF4-DDIT3-TRIB3-AKT-mTOR axis	Kuwanon H (KuH) [96]
	Upregulation of C/EBP homologous protein (CHOP)	Acetylsalicylic acid (ASA) and salicylic acid (SA) [97]
	Upregulation of eIF2α, CHOP, and caspase-3	Resveratrol [98], Shikonin [99]
	Inhibition of the AKT/GSK3-β/β-catenin pathway	Tumor suppressor candidate 3 (TUSC3) [101]
	Inhibition of MITF and β-catenin pathways	Honokiol [102]
	Activation of the IRE1α/Xbp1 pathway	Pinocembrin [103]
	Upregulation of ROS generation	Luteolin [104]
	Inhibition of the BRAF and RAF/MEK/ERK (MAPK) pathways	Vemurafenib with binimetinib [105]
	Upregulation of eIF2α	Bornyl cis-4-hydroxy cinnamate [106]
	Inhibition of GRP78 and upregulation of CHOP	25-epi Ritterostatin GN1N [107]
	Activation of the PERK/p-eIF2α/ATF4/CHOP pathway	Vitamin E δ-tocotrienol (δ-TT) [108]
	Binding of hnRNP H1 and H2	Novel Pyrrolidine Diketopiperazines 2155–14 and 2155–18 [109,110,111]
Autophagy	Upregulation of BECN1 by miR1290 inhibition	Polygonatum odoratum lectin (POL) [115]
	Activation of the JNK pathway	miR-24-1-5p [116]
	Activation of HIF-1α/BNIP3/BNIP3L pathway	Proopiomelanocortin (POMC) [117]
	Accumulation of damaged mitochondria via blocking the mitophagic flux to lysosomes	siTRPML1 [118]
	Downregulation of LC3, ERK1/2 and MITF	Combination of doxycycline and minocycline [119]
	Autophagosome accumulation	Sasanquasaponin ΙΙΙ [121]
	Regulation of the CXCR4/CXCL12 axis	Liposomes containing paclitaxel and hydroxychloroquine [122]
Necroptosis	Inhibition of mitochondrial complex I-mediated cellular ROS level increase	BAY 87-2243 [127]
	Upregulation of death receptors and scaffold protein by activating caspase-8	10-Methoxy-1,2,3,4-tetrahydrobenzo(g) (1,3) diazepino(1,2-a)-(1,8) naphthyridin-6-yl) (phenyl) methanone (3u) [128]
	Activation of caspase-3, caspase-9, and poly (ADP-ribose) polymerase 1	Evodiamine (EVO) [131]
	Activation of the RIPK1/RIPK3/MLKL pathway	Protein-bound polysaccharides (PBPs) [133]
	Increasing levels of CHOP and RIP1	Shikonin [134]
	Increasing the cellular level of ROS	Pleuromutilin derivative compound **38** [132]
Pyroptosis	Activation of the Tom20-Bax-caspase-3-GSDME pathway	CCCP and several chemotherapeutic drugs, such as SSZ [137]
	Targeting CXCR4	A self-assembling nanotoxin (T22 peptide) [138]
	Activation of caspase 3	Raptinal [139]
	T-cell accumulation/activation, GSDM E cleavage, and release of HMGB1	PLX4720/PD0325901 [142]
	Activation of caspase 3 and GSDM E/D cleavage	Temozolomide/chloroquine [140]
	Activation of gasdermin	Phe-BF3 [143]
Ferroptosis	GPX4 inhibition	BET inhibitor [145],
	IFN-γ release and driving action	nanoparticle of miR-21-3p [146]
	Upregulation of GOT1	anti-miR-9 [147]
	SLC1A5-mediated glutamine uptake and MDA accumulation	anti-miR-137 [148]
	Inhibition of the AKT/GPX4 pathway	ARG2 KO [149]
	Inhibition of NEAT1	Gambogenic acid (GNA) [150]
	Activation of the AMPK/mTOR pathway	Arachidonate 5-lipoxygenase (ALOX5) [151]
	Inhibition of USP14	MitoCur-1 [152]
	GPX4 inhibition-mediated metabolic reprogramming	DET and DETD-35 [153]
	Upregulation of SVCT2	Blue light [155]
	Increasing lipid peroxidation	Gallic acid (GA) with low-level laser [156],protoporphyrin IX-based polysilsesquioxane nanoparticles. (PpIX-PSilQ NPs) [157]
	Inhibition of the GSK3β-mediated Keap1/Nrf2/HO-1 pathway	Nobiletin [158]
Cuproptosis	Inhibiting the MAPK signaling pathway	Chelating copper [162]
